# Human Origins and the Search for “Missing Links”

**DOI:** 10.1371/journal.pbio.1001333

**Published:** 2012-05-15

**Authors:** Johannes Krause

**Affiliations:** Institute for Archaeological Sciences, University of Tuebingen, Tuebingen, Germany

## Abstract

The study of human evolution is filled with exciting discoveries, contentious disputes, and immense promise. Johannes Krause reviews John Reader's book on the history of paleoanthropology.

**Figure pbio-1001333-g001:**
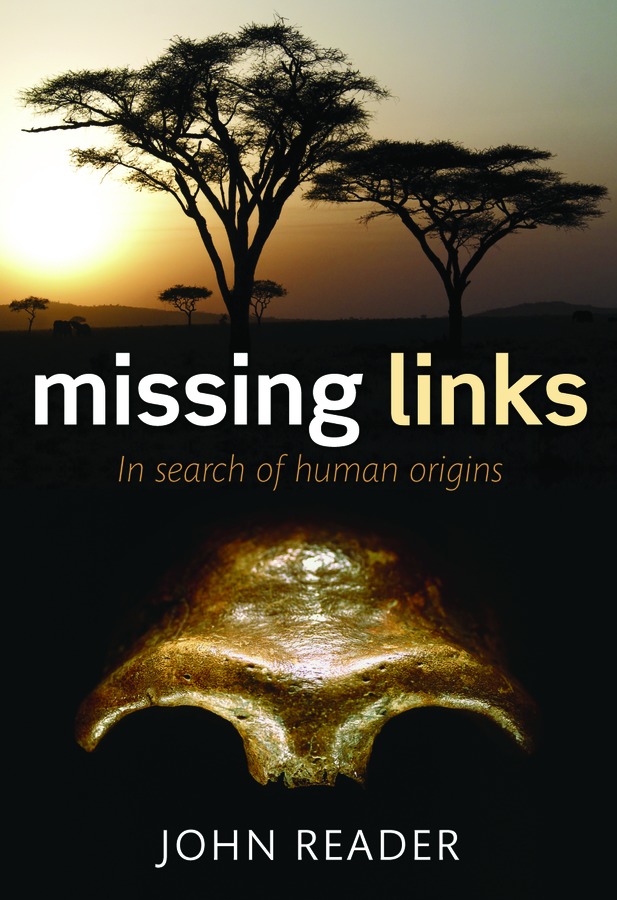
Reader J (2011) Missing Links: In Search of Human Origins. New York: Oxford University Press. 350 p. ISBN-13: 978-0199276851 (hardcover). US$34.95.

[Fig pbio-1001333-g001]Humans are naturally fascinated by questions concerning our own origin, not just where we come from but what made us the way we are. In almost all cultures and religions one finds some form of creation myth explaining how their tribe or people came into existence, ranging from the Mayan god Heart-of-Sky that after several failed attempts finally made the true men from maize to the biblical god that created man from wet clay and women from Adam's rib. Yet it wasn't until the middle of the 19th century that the scientific debate about the origin of our species took off, sparked by a single short hint in *On the Origin of Species* by Charles Darwin that challenged the broadly accepted view of the time that humans were created by a divine entity. Darwin's work implied that humans are not an exception to the processes that drive evolution, such as natural selection, but rather that we evolved from primate ancestors over millions of years, leaving behind a number of extinct ancestral forms.

In 1856, three years before Darwin published his book, the first evidence for such ancestral human forms was brought forward, with the discovery of a human fossil in the Neander Valley in Germany by quarry workers. Hand in hand with Darwin's book, the Neandertal fossil started a heated debate about the evolution of our species that continued over several decades before more evidence in the form of human fossils was discovered.

By now, more than 1,000 human fossils have been found spanning the last 7 million years of our evolution. They give scientists the chance to reconstruct the ancestral stages of our lineage and to define various important changes in our morphology during the course of our evolution. But despite the large number of remains, the reconstruction of the human family tree is far from trivial. Over the last century it became clear that human evolution follows no straight line leading from a common ancestor with chimpanzees to contemporary modern humans. Instead, our ancestry is rather a thicket of branches, with several species co-existing at any given time, where we anatomically modern humans are the sole survivors.

The discovery of a yet unknown human fossil, therefore, still receives tremendous attention from the media and public and is often presented as shining a new light on our evolution, questioning previous interpretations, and presenting a new link between various branches of our evolutionary tree. Such fossils are often referred to as “missing links”, because they present the first physical evidence that lends credibility to previous theoretical speculations about our past.

In his new book, *Missing Links: In Search of Human Origins*, the author John Reader gives a detailed account of the often exciting stories behind the discoveries of some of the most important human fossils considered missing links at the time of their discovery, ranging from the first Neandertal specimen discovered in the 1850s to the recently discovered enigmatic “Hobbits” (*Homo floresiensis*) unearthed in 2003 on the island of Flores in Indonesia. In an engaging way, Reader presents the fragmentary fossil evidence that slowly emerged over time and the competing interpretations that were built upon them. He begins by providing the historical background of the science that would eventually become paleoanthropology, the study of human evolution from fossil evidence, by describing 500 years of scientific discovery that led to the realization that our planet is in fact billions of years old and that life evolved in various steps. As a result, most life forms are extinct today, leaving only fossils behind as evidence of their existence, including our own ancestors.

The book provides an excellent introduction to the history of paleoanthropology, covering the most important fossils and the scientists who heavily influenced our understanding of human origins by their interpretation of the fragmentary fossil record. Reader furthermore provides the background on some of the more controversial episodes of paleoanthropology, such as the Piltdown hoax, a human fossil that puzzled anthropologists over decades after its discovery at the beginning of the last century. The fossil displayed ape-like facial features and a human-like brain case, suggesting that the big brain evolved first in our evolution. It was shown, however, that the fossil was artificially constructed by a very skilled person who managed to delude some of the best scientists in the field during that time. Another unfortunate episode covered by Reader is the tragic loss of the famous Peking Man fossils in China, the largest collection of *Homo erectus* fossils ever found, in the jumble of World War II. Using dozens of beautiful color images that Reader took himself over the more than 30 years of his career, he provides almost intimate portraits of the human fossils and their discoverers.

As is often said, paleoanthropology is probably the only research field that has more scientists than study objects, which inevitably contributes to restricted access to the fossils, and various re-interpretations of existing hypotheses that are often in the absence of new conclusive evidence. Reader astutely notes the highly political nature of the field, where the merit of a given hypothesis “becomes more a matter of personalities than science”. Few fields of research are subject to so many competing hypotheses, as illustrated by the variable number of ancestral species assigned to the human lineage by different authors, ranging from four to a maximum of 25. Reader, however, maintains an impartial voice in his explanations of the various controversies and displays no personal agenda in the debates, as is otherwise often the case in popular books on this topic; instead, he provides an evenhanded account of the evidence, leaving room for his audience to draw their own interpretations.

As Reader notes, it is not always hard work and dedication that leads to exciting new fossil discoveries, but often sheer luck. Certainly there are other fields where chance and even carelessness lead to exciting discoveries, as illustrated by Alexander Flemming, who forgot to dispose of petri dishes, only to notice a bacteria-killing mold: penicillin. What distinguishes paleoanthropology, however, is that a paleoanthropologist retains a certain ownership over the fossil he discovers. Many anthropologists have thus based their entire career on a single site or finding, allowing access to only a limited circle of people that support their own ideas, creating a biased interpretation and often controversy over the interpretation of the fossil evidence. Reader gives several rather tragic examples of scientists who were criticized or dismissed by their colleagues, such as Eugene Dubois, who discovered Java Man at the end of the 19th century, at the time of discovery the oldest homo fossil found and later named *Homo erectus*, or Raymond Dart, who discovered the Taung Child (*Australopithecus africanus*) in the 1920s, presenting the first evidence that the early evolution of humans had started in Africa rather than Asia. The book gives further examples of heated discussions between Donald Johanson and Richard Leakey in the late 1970s over the interpretation of some of the African fossils such as the famous Lucy. But as Reader states towards the end of his book, “[u]ltimately, as the short story of paleoanthropology repeatedly demonstrates, it is evidence, not eloquence that settles the argument”.

From the vantage point of a geneticist, where data are commonly deposited into publically available electronic databases, it is difficult to comprehend the politics of restricted access to fossils. However, times are rapidly changing in the field of paleoanthropology, with the possibility of creating virtual images of fossils using high resolution computer tomography and 3D printing. Morphological data can be made publically available and thus can be shared between researchers worldwide. Furthermore, this eliminates the requirement of having irreplaceable fossils jet set around the world to different laboratories, allowing them to safely stay in the countries where they were discovered. This will further exert a greater political ownership to the countries where the fossils were discovered rather than to individual scientists, in contrast to the massive looting of archaeological material that occurred historically.

A further advantage of digitalized virtual data is the ability for large-scale computational comparisons using hundreds of fossil and modern specimens. This so-called morphometric approach is growing in popularity amongst paleoanthropologists, and allows prospective and retrospective simulations of evolutionary changes such as brain development. This method offers reproducibility and greater scientific rigor over traditional analyses of fossil shape that were largely descriptive and restricted in scope.

In addition to the significant impacts of new dating techniques and morphometrics, Reader extends his discussion to biomolecular analyses of fossils and their tremendous influence on paleoanthropology over the last two decades. The analysis of stable carbon and nitrogen isotopes first applied on collagen from fossil human bones in the 1990s provided the opportunity to study the resource exploitation by various hominin groups. In the case of Neandertals, it was found that they had an extremely high protein intake, comparable to that of carnivores.

Other insights from the molecular lab came from the field of genetics. The late 1980s brought us the first worldwide genetic dataset of mitochondrial DNA (mtDNA) sequences from various contemporary human populations. It became clear that the largest diversity of human mtDNA exists today in Africa. All people outside of Africa were found to share a recent common ancestor that left Africa around 50,000 years ago, giving credibility to the still hotly debated “Out of Africa” hypothesis of modern human evolution proposed by several paleoanthropologists in the mid-1980s.

In the mid-1990s, the mtDNA of the Neandertal-type specimen was analyzed, the very fossil that had prompted the debate on human origins in the 1850s. It turned out that the Neandertal mtDNA was different from that of modern humans, with a divergence time of roughly 500,000 years before present, lending additional support to the Out of Africa theory.

The biggest revolution, however, was yet to come. Recent years have seen not only mtDNA sequences from about a dozen Neandertals spanning their entire range from the Spanish Peninsula to southern Siberia, but new sequencing technologies have made it possible to reconstruct entire genomes from well preserved archaic human fossils. The first such genome presented in 2010 was a composite genome from several Neandertals, providing further support for the Out of Africa hypothesis, with evidence of only small amounts of genetic admixture from Neandertals into all non-African modern humans, in the range of 2.5% genetic ancestry. The second ancient archaic genome was an even bigger surprise. It derived from a tiny finger bone unearthed in 2008 in the Denisova cave in Siberia. The DNA from that bone suggested that the finger derived from an archaic human population that is more closely related to Neandertals than to anatomically modern humans, yet it is quite distinct, showing several archaic and derived features unique to this population of hominins. The population represented by this tiny bone was therefore named “Denisovan” after the place of discovery, analogous to the naming convention of “Neandertals”. The comparison to contemporary modern humans furthermore revealed that the Denisovans genetically admixed with modern human populations from Southeast Asia and Australia, suggesting that this archaic hominin had an immense range over large parts of East Asia from southern Siberia down to the eastern islands of Indonesia. Thus, the first evidence of a “missing link” was in fact provided by genetics, introducing us to one of our close ancestors that roamed large parts of East Asia before we appeared on the scene, for which a more complete fossil has yet to be discovered. The close genetic relationship of Denisovans and Neandertals provisionally rules out other Asian fossils such as *Homo erectus* and the Indonesian “Hobbits” as potential candidates for what Denisovans might have looked like; however, other fossils from China and parts of Asia might eventually be linked or discovered that will give us a full anatomical picture of the “missing link” Denisovan.

It is thus hopeful that this second volume of “missing links” by Reader is not the last edition, and that future discoveries as well as new analysis techniques and tools will help us to further illuminate the muddle that we call our ancestry.

**About the Author**

Johannes Krause is Professor for Paleogenetics at the Eberhard Karls University in Tuebingen, Germany. He is specialized in ancient DNA research. Before joining the faculty in Tuebingen he worked in the Max Planck Institute for Evolutionary Anthropology in Leipzig, Germany, where he was one of the leading scientists deciphering the Neandertal genome. In addition, he found the first genetic evidence of a fossil human form that was named Denisovan after the place of discovery in a cave in the Altai Mountains in southern Siberia. This so far unknown hominin roamed large parts of East Asia during the late Pleistocene before the arrival of modern humans. Professor Krause furthermore pioneered genome-wide analysis of ancient pathogens by deciphering the genome of medieval *Yersinia pestis*, the bacteria that caused the Black Death in the 14th century.

